# A Novel, Hybrid-Integrated, High-Precision, Vacuum Microelectronic Accelerometer with Nano-Field Emission Tips

**DOI:** 10.3390/mi9100481

**Published:** 2018-09-20

**Authors:** Haitao Liu, Kai Wei, Zhengzhou Li, Wengang Huang, Yi Xu, Wei Cui

**Affiliations:** 1 School of Microelectronics and Communication Engineering of Chongqing University, Chongqing 400044, China; 201712131047@cqu.edu.cn; 2 Chongqing acoustic optoelectronic Co. Ltd. of China Electronics Technology Group Corporation, Chongqing 400060, China; hwg_cq@163.com (W.H.); Cuiwei@analogfoundries.com (W.C.); 3 National Key Discipline Laboratory of Novel Micro/Nano Devices and Systems Technologies, Chongqing University, Chongqing 400044, China; xuyibbd@cqu.edu.cn

**Keywords:** field emission, hybrid integrated, vacuum microelectronic, cathode tips array, interface ASIC

## Abstract

In this paper, a novel, hybrid-integrated, high-precision, vacuum microelectronic accelerometer is put forward, based on the theory of field emission; the accelerometer consists of a sensitive structure and an ASIC interface (application-specific integrated circuit). The sensitive structure has a cathode cone tip array, a folded beam, an emitter electrode, and a feedback electrode. The sensor is fabricated on a double-sided polished (1 0 0) N-type silicon wafer; the tip array of the cathode is shaped by wet etching with HNA (HNO_3_, HF, and CH_3_COOH) and metalized by TiW/Au thin film. The structure of the sensor is finally released by the ICP (inductively coupled plasma) process. The ASIC interface was designed and fabricated based on the P-JFET (Positive-Junction Field Effect Transistor) high-voltage bipolar process. The accelerometer was tested through a static field rollover test, and the test results show that the hybrid-integrated vacuum microelectronic accelerometer has good performance, with a sensitivity of 3.081 V/*g*, the non-linearity is 0.84% in the measuring range of −1 *g*~1 *g*, the average noise spectrum density value is 36.7 μV/Hz in the frequency range of 0–200 Hz, the resolution of the vacuum microelectronic accelerometer can reach 1.1 × 10^−5^ *g*, and the zero stability reaches 0.18 mg in 24 h.

## 1. Introduction

There is an increasing demand for small-sized, lightweight, and low-powered sensing systems in micro-accelerometers. Especially, MEMS (micro-electromechanical system)-based accelerometers find great applications in navigation systems [1,2,3,4], inertial sensors [5,6,7], seismometers [8], space microgravity [9], military affairs [10], and optical devices [11]. Since the world’s first field-based sensor was launched, field emission devices have been widely used due to their high accuracy, high sensitivity, and anti-radiation advantages.

The vacuum microelectronics accelerometer is based on the field emission principle [12]; while field emission has two distinct advantages over other accelerometers, due to the feature of cold cathode emission [13,14,15], the output current signal of the sensor changes exponentially with the acceleration, so that the sensitivity is very high, and the current output of the sensor makes its interface current relatively simple. In this paper, a novel, hybrid-integrated, high-precision vacuum microelectronic accelerometer is proposed. This paper presents the principle and structure, and the design and fabrication of the sensor and interface circuits; then, it demonstrates the experimental results of the circuits and the accelerometer, and the conclusion is finally reported.

## 2. Principle and Structure

The vacuum microelectronics accelerometer is based on the metal field emission principle that metal field emission is a kind of electron emission phenomenon that relies on a strong external electric field to suppress the potential barrier of the metal surface, reduce the barrier, and narrow the barrier width. With the increasing strength of the applied electric field, the height of the surface barrier is not only reduced but the width is also narrowed. When the barrier width is narrow enough to be comparable to the electron wavelength, the electrons can escape through the potential barrier, thereby forming a field electron emission in the vacuum.

The metal field emission current can be calculated by the Fowler and Nordheim Formula (F–N) [16,17]. The simplified Fowler–Nordheim Formula is shown as Equation (1):

(1) $$J=1.5\times 10^{-6}\frac{E^{2}}{\emptyset }\exp\left(\frac{10.4}{\emptyset^{1/2}}\right)\exp(-16.44\times 10^{7}\frac{\emptyset^{3/2}}{E})$$

in which *J* is the field emission current density (A/cm^2^), *E* is the surface electric field strength (V/cm), and ∅ is the work function (eV).The surface electric field strength of a tip is shown as Equation (2):

(2) $$E=\frac{2}{rln(\frac{d}{r})}V$$

in which *r* is the curvature radius of the cone tip, *d* is the distance between the cathode cone and the anode plate, and *V* is the voltage applied on the anode.

**Figure 1:**
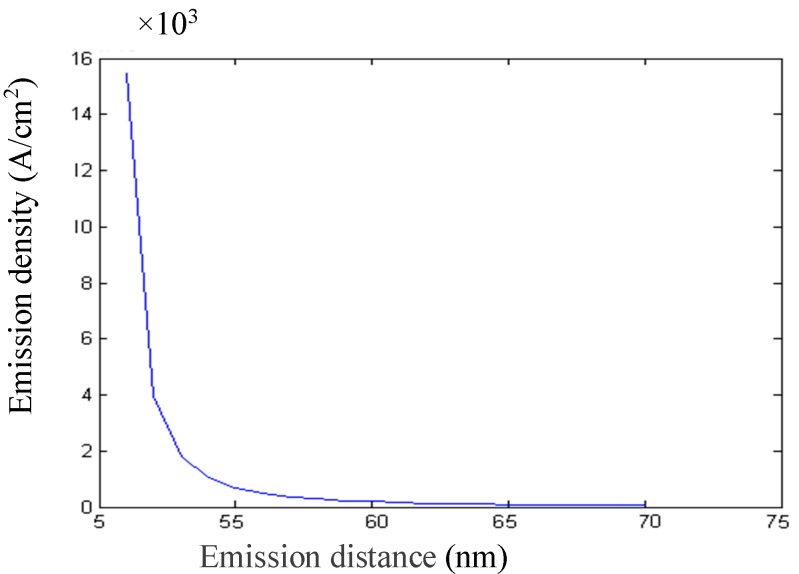
Emission current density vs. distance between the cathode tips and the anode.

**Figure 2:**
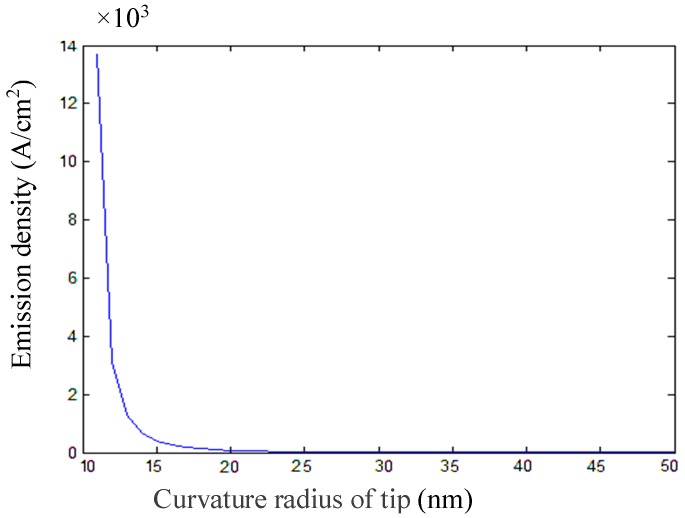
Emission current density vs. the curvature radius of the tip.

The distance between the emission current density and the cone tip radius of curvature, and the distance between the cathode cone and the anode plate, can be obtained by calculation, as shown in Figure 1 and Figure 2. Figure 1 shows that the emission current density decreases exponentially, with the distance from the tip to the anode plate being at a certain emission voltage, and Figure 2 shows that the emission current density decreases with the radius of curvature of the cone tip. In order to obtain a larger emission current at a smaller operating voltage, the radius of curvature of the tip and the distance from the tip to the anode plate should be smaller.

**Figure 3:**
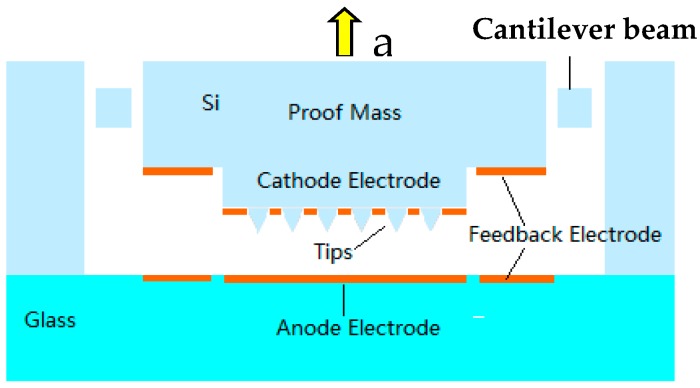
Structure of the vacuum microelectronic accelerometer.

The structure of sensor consists of a proof mass with a field emission cathode tip array, a cantilever beam, an anode, and feedback electrodes, as shown in Figure 3. There are about 10,000 tip arrays on the cathode, which makes the emission current increase greatly. The external acceleration will lead to the proof mass moving, and this will cause the distance between the cathode and the anode tip array to change, causing the cathode field emission current to dramatically vary with an exponential relationship, so that the acceleration can be obtained by detecting the cathode emission current.

## 3. Design and Simulations

### 3.1. The Sensor-Sensitive Unit

**Figure 4:**
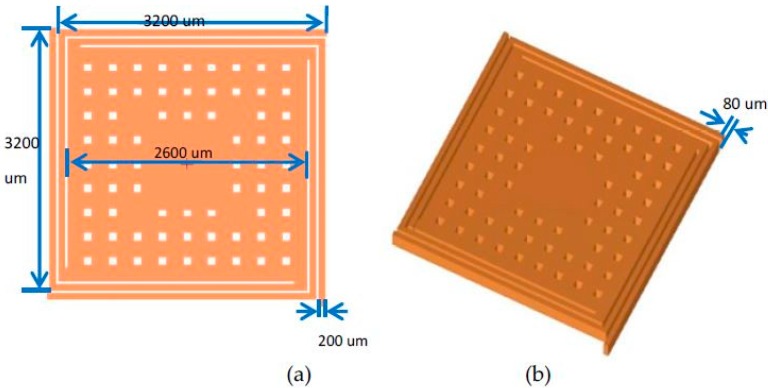
Structure of the sensitive structure of the vacuum microelectronic accelerometer. (**a**) Plan view of the sensitive structure; (**b**) 3D map view of the sensitive structure.

The sensitive element was designed by a cantilever-mass, and the cantilever was a novel folded beam structure, as shown in Figure 4. Based on the deformation energy analysis method, and mechanical and electric analyses, the rectangular folding beam elastic stiffness of the vacuum microelectronic accelerometer was calculated [18]. The design parameters of the structure are shown in Table 1.

**Figure 5:**
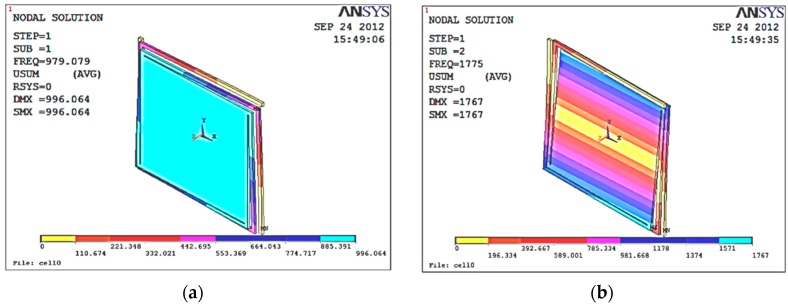

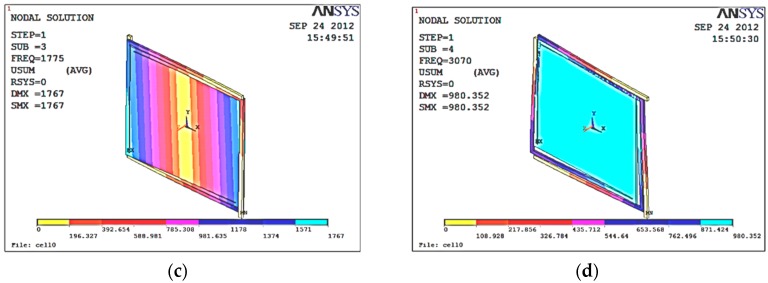
Modal graph of the vacuum microelectronic accelerometer. (**a**) The first mode, (**b**) the second mode, (**c**) the third mode, and (**d**) the fourth mode.

The resonant frequency and modal response of the sensor were analyzed by finite element simulation (FEM); the analysis results by ANSYS are shown in Figure 5 and Table 2. Figure 4a shows the first mode in the working mode of the accelerometer; the resonant frequency was 979.08 Hz. Figure 4b shows the second mode, in which the proof mass rotates around a horizontal axis, and the resonant frequency was 1774.5 Hz. The third mode was the same as the second mode, only that the rotation axis was different, and the resonant frequency was also 1774.7 Hz, as shown in Figure 4c. Figure 4d shows the fourth mode in which the support beams vibrate, and the resonant frequency was 3069.8 Hz. The frequency of the interfering modes was far from the operating mode.

**Figure 6:**
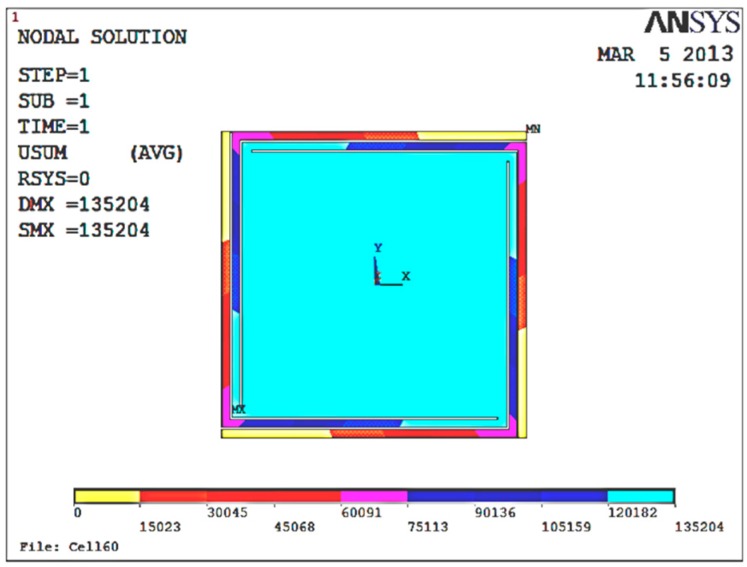
Displacement contour under +1 *g* acceleration.

The elastic stiffness *K* can be calculated by Equation (3):

(3) $$K=\frac{ma}{x}$$

in which *m* is the quality of the proof mass, *a* is the acceleration, and *x* is the moving distance of the proof mass. Figure 6 shows the results of the static force analysis of the sensor; the displacement as represented by the color from the left to the right of the figure increases in turn. The simulation analysis results show that the displacement of the proof mass reached the maximum value of 0.13 μm when there was ± 1 *g* of acceleration along the *Z* axis; the quality of the proof mass was 4.72 × 10^−6^ kg; and the effect force applied on the proof mass was 4.72 × 10^−6^ N, so that the approximate elastic stiffness was 36 N/m by calculation. 

### 3.2. Process and Fabrication

**Figure 7:**
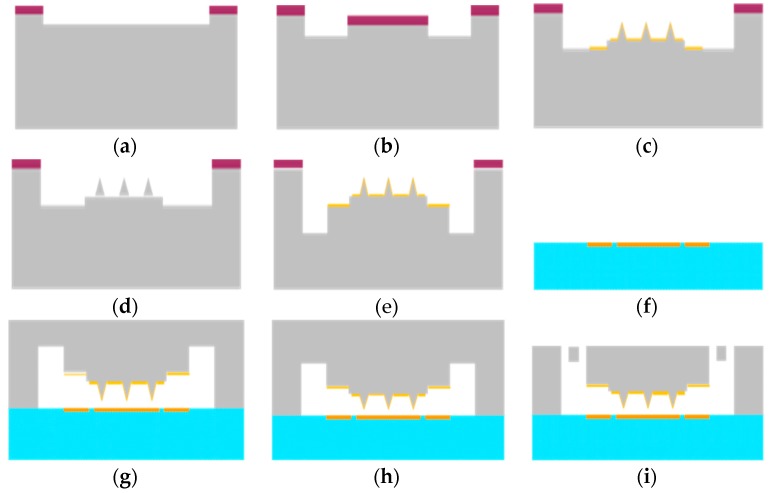
Process of the vacuum microelectronic accelerometer. (**a**) Etching groove windows to form a bonded anchor. (**b**) Etching groove windows to form the cone station. (**c**) Corrosion cone formation and sharpening. (**d**) After ion implantation, cone metallization is performed. (**e**) ICP etching of the front to form the beam area. (**f**) Growth of the electrodes on glass. (**g**) Bonding the silicon and glass. (**h**) The silicon is thinned by KOH etching. (**i**) ICP structure release to form the beam.

The vacuum microelectronic accelerometer is based on bulk silicon MEMS technology, which is based on the double-side polished N-type (1 0 0 direction) silicon wafer, which has a high tensile strength and low mechanical losses. The process of the fabrication of a vacuum microelectronic accelerometer includes the silicon process and the glass process, as shown in Figure 7. During the wafer process, etching groove windows are made to form a bonded anchor (a); etching groove windows are made to form the cone station (b), followed by corrosion cone and sharpening (c), and cone metallization after ion implantation (d); finally, ICP etching is performed to form the beam area (e). The glass processes form the electrodes on the glass surface (f). Finally, the silicon and glass are bonded (g), and the silicon is thinned by KOH etching (h); the ICP structures are then released to form the beam (i). After all these processes, the sensitive unit of the vacuum microelectronic accelerometer is formed.

**Figure 8:**
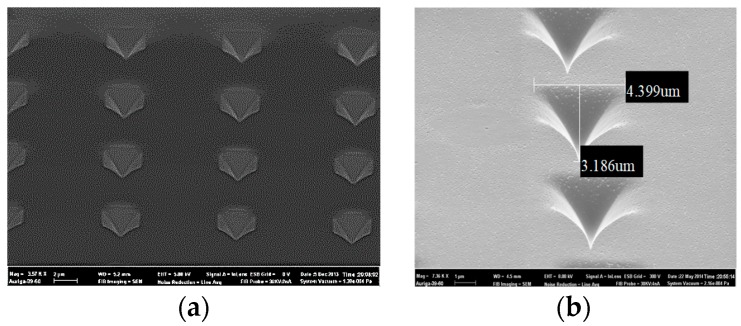

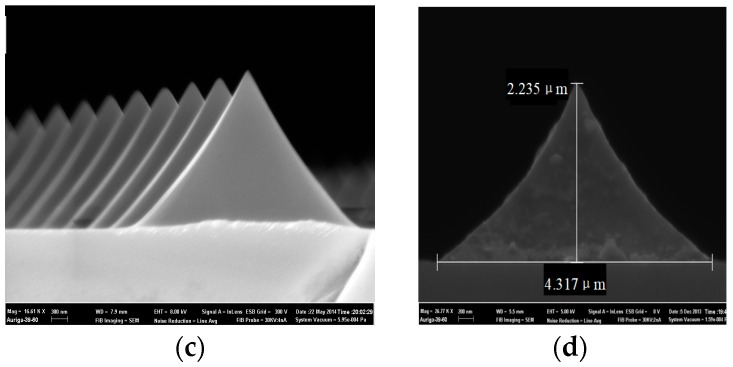
Scanning electron microscopy (SEM) of the cathode tip array. (**a**) Array front view, (**b**) partial front view, (**c**) array side view, and (**d**) partial side view.

The tip shape is one of the main factors that affect the performance of the accelerometer, and the processes of the tip is also one of the key processes for the accelerometer. The silicon tip arrays are formed by wet etching with the HNA solution (HNO_3_, HF, and CH_3_COOH) and metalized by TiW/Au thin film; the morphology of the tip is an ideal pyramid, as shown in Figure 8.

The cathode cone tip surface of the vacuum microelectronic accelerometer is easily oxidized under a low vacuum, which leads to an emission current drop and instability. In order to improve the emission current stability of the vacuum microelectronic acceleration sensor, the surface was coated with a layer composite metal film to protect the cone tip, which can effectively improve the stability of the emission current.

### 3.3. Interface Circuit

**Figure 9:**
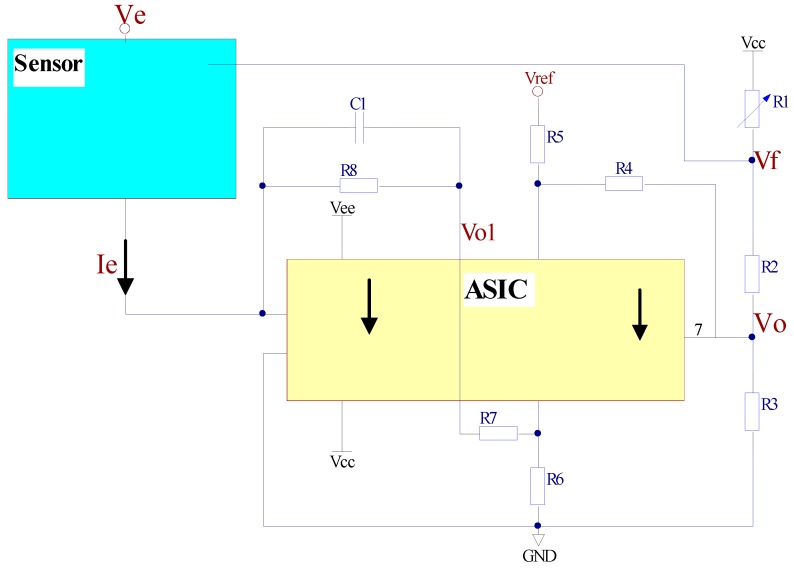
Schematic of the interface circuit of the vacuum microelectronic accelerometer.

The interface circuit includes a current–voltage conversion, a differential amplifier circuit, and electrostatic force feedback circuit modules, as shown in Figure 9. The mass will produce a slight displacement under acceleration, and it will lead to changes in the emission current; after the current is converted into voltage and amplified, the voltage signal *V_o_* associated with the displacement of the mass is obtained.

The transmission current of the cone is exponentially related to the change of the displacement. When the displacement change is very small, the current can be approximated linearly. The voltage after the *I*–*V* conversion is calculated by Equation (4):

(4) $$V_{o1}=R_{8}\cdot I_{e}\left(1-\alpha \Delta x\right)$$

When the resistance *R*_5_ = *R*_7_ and *R*_4_ = *R*_6_, the output voltage *V_o_* after the differential amplification is calculated by Equation (5).

(5) $$V_{o}=\frac{R_{4}}{R_{5}}\left(V_{o1}-V_{ref}\right)$$

In order to improve the output linearity and the dynamic response range of the vacuum microelectronic accelerometer, the linear feedback network was designed by the electrostatic force balance technique. The output voltage *V_f_* of the feedback circuit is

(6) $$V_{f}=\frac{R_{2}+R_{3}}{R_{1}+R_{2}+R_{3}}\times V_{cc}+\frac{R_{1}}{R_{1}+R_{2}}\times V_{o}$$

**Figure 10:**
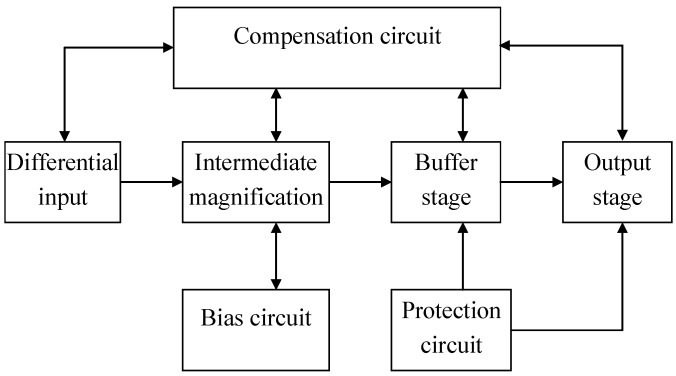
Basic structure of the interface ASIC circuit.

**Figure 11:**
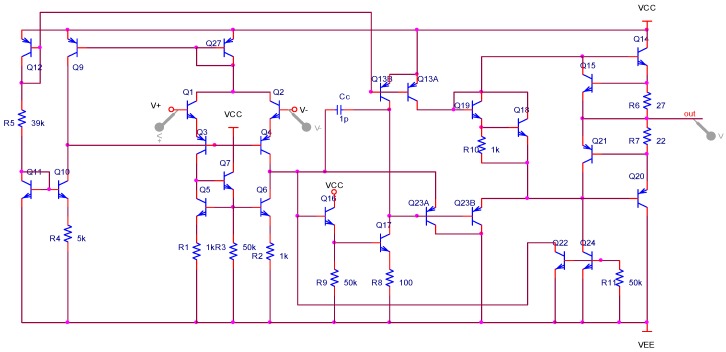
Schematic circuit of the ASIC.

In order to improve the signal-to-noise ratio of the circuit, the two op amps, U1A and U1B in the circuit, were designed as application-specific integrated circuits (ASICs), which include an integrated dual operational amplifier. The basic structure of the interface ASIC is shown in Figure 10 and Figure 11 is the schematic circuit of the interface ASIC.

**Figure 12:**
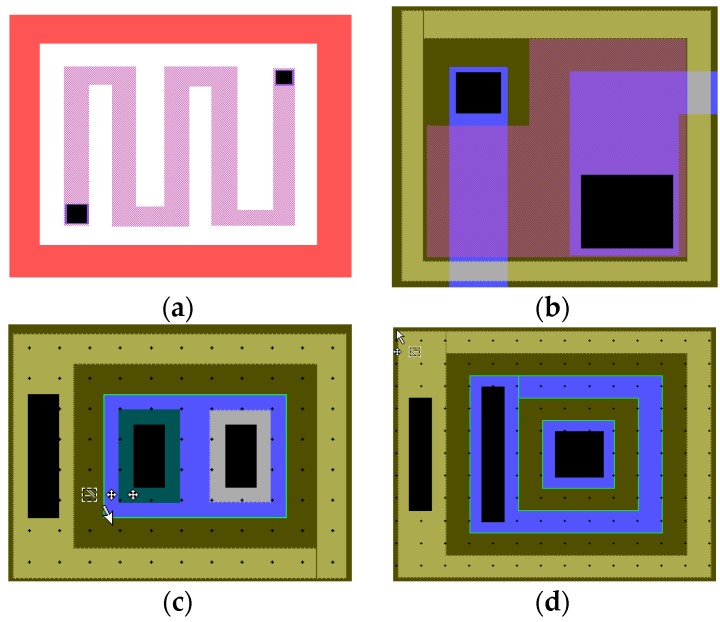
Layout of the basic component. (**a**) Resistor unit layout, (**b**) MOS (Metal Oxide Semiconductor) capacitor layout, (**c**) NPN (Negative Positive Negative) transistor layout, and (**d**) Substrate lateral PNP transistor layout.

Finally, the ASIC was fabricated based on the P-JFET high voltage bipolar process in the Chongqing acoustic optoelectronic Co. Ltd. of China Electronics Technology Group Corporation. Figure 12 shows the layout of the basic component units of the ASIC interface.

## 4. Tests and Results

**Figure 13:**
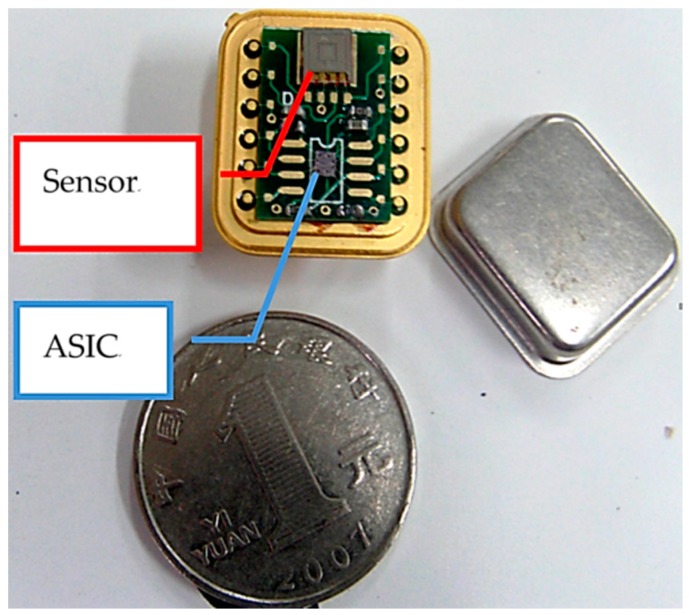
Photo of the hybrid integrated vacuum microelectronic accelerometer.

Finally, the sensitive structure unit and the interface ASIC chip were integrated on a PCB board, and the accelerometer was packed in the vacuum packaging machine, whose vacuum degree could reach 10^−4^ Pa; getter was added into the tube to maintain a high vacuum degree for a long time. The photo of hybrid integrated vacuum microelectronic accelerometer is shown in Figure 13. The working voltage of the accelerometer was a ±15 V power supply.

Table 3 shows the test results of main parameter of the interface ASIC. The offset voltage *V_os_* was −1.29 mV; the offset current *I_os_* was −0.5 nA; the common-mode rejection ratio CMRR was 81 dB; the voltage gain open-loop differential mode voltage gain, *A_vo_*, was 103 dB; the power supply rejection ratio, PSRR, was 106 dB; and all these parameters showed that the interface ASIC has very good performance indicators, and that it can realize the signal amplification and the processing of the sensor.

**Figure 14:**
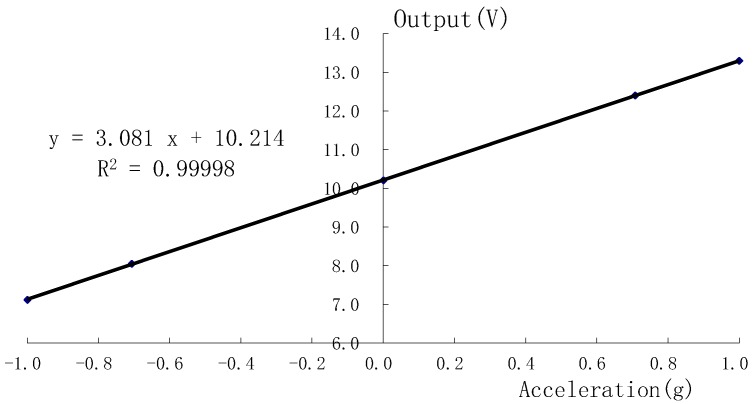
Curve of the output data vs. input data.

A gravitational field static rollover test was carried out in order to test the sensitivity, linearity, and zero stability, to use the mirroring precision rotary indexing head. The testing data are shown in Figure 14 and Table 4. Test results showed that the measuring range was −1 *g*~1 *g*, and that the sensitivity of the accelerometer was 3.081 V/*g*; the least squares fitting correlation coefficient reached 0.99998, and the non-linearity was 0.84%.

**Figure 15:**
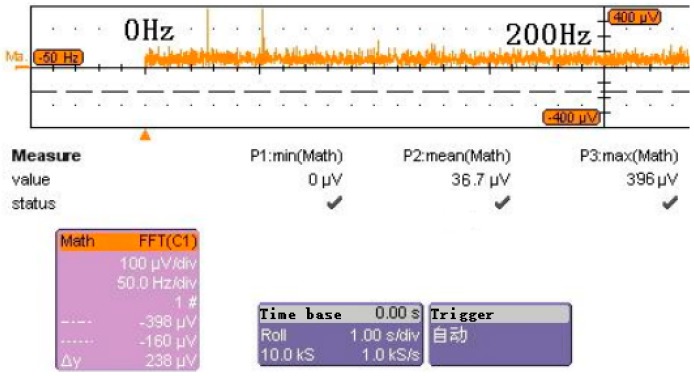
Noise spectrum density of the vacuum microelectronic accelerometer.

Figure 15 shows the spectrum density of output signal, in which the x-axis is the frequency and the y axis is the peak-to-peak spectrum density (μV/Hz), and the average noise spectrum density value is 36.7 μV/Hz in the frequency range of 0–200 Hz. Because the output sensitivity of the accelerometer is 3.081 V/*g*, the resolution of the vacuum microelectronic accelerometer can reach 1.1 × 10^−5^ *g*.

**Figure 16:**
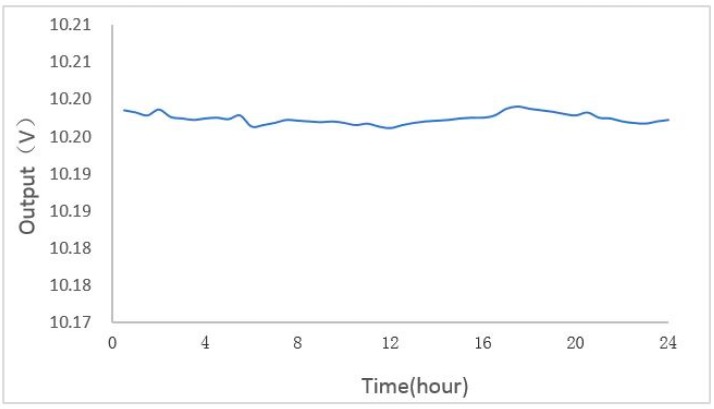
Zero stability of accelerometer in 24 h.

The accelerometer is placed at the zero acceleration position, and the output value is measured every 0.5 h. The output value of the accelerometer is recorded in 24 h to calculate its bias stability shown in Figure 16; the result shows that the zero stability reaches 0.18 m*g* in 24 h.

## 5. Conclusions

In this study, we presented a novel hybrid integrated vacuum microelectronic accelerometer. The structure and the working principles of the sensor were studied in detail, and the mechanistic characteristics of the sensitive structure were analyzed by finite element analysis. Furthermore, the fabrication process and the interface ASIC circuits were designed.

Because of the optimized design of the structural design and process while improving the cone tip production process, the most critical point of the vacuum microelectronic accelerometer is that the interface circuit was designed based on the application-specific integrated circuit, so that the system’s signal-to-noise ratio was improved greatly. The test results of the vacuum microelectronic accelerometer show that the sensitivity is about 3.081 V/*g*, the nonlinearity is about 0.84% over a range of −1 *g*~1 *g*, the average noise spectrum density value is 36.7 μV/Hz in the frequency range of 0–200 Hz, the resolution of the vacuum microelectronic accelerometer can reach 1.1 × 10^−5^ *g*, and the zero stability reaches 0.18 mg in 24 h. This can be widely applied in high-precision inertial systems and other similar applications, such as those for acoustic measurement, navigation, earthquake monitoring, aerospace, and so on.

There are also some problems that need to be resolved; for example, performance should be greatly boosted if the sensitive structure and interface ASIC are monolithic integrated on one chip, along with chip-level vacuum packaging.

## Figures and Tables

**Table 1:** Key structure parameters of the sensor.

Parameters	Design Value
Beam Length	3200 µm
Beam Width	200 µm
Beam Thickness	80 µm
Mass Side Length	2600 µm
Mass Thickness	80 µm

**Table 2:** Mode analysis results.

Set	Freq (Hz)	Loadstep	Substep	Cumulative
1	979.08	1	1	1
2	1774.5	1	2	2
3	1774.7	1	3	3
4	3069.8	1	4	4

**Table 3:** Main parameters of the ASIC.

Parameters	*V_os_* (mV)	*I_b+_* (nA)	*I_b−_* (nA)	*I_os_* (nA)	*A_vo_* (dB)	CMRR (dB)	PSRR (dB)
Design specifications	<5	<150	<150	<30	>96	>70	>65
Test Results	−1.29	−16.3	−15.8	−0.5	103	81	106

**Table 4:** Datum of the static gravitational field roll test.

Acceleration (*g*)	Measure Value (V)	Fitting Value (V)	Deviation Value (V)	Non-Linearity
+1.0	13.292	13.314	−0.022	−0.71%
+0.7	12.397	12.411	−0.014	−0.46%
0	10.206	10.230	−0.024	−0.76%
−0.7	8.053	8.049	0.004	0.12%
−1.0	7.120	7.146	−0.026	−0.84%

## References

[1] Syed Z., Aggarwal P., Niu X., Sheimy N. (2007). Economical and robust inertial sensor configuration for a portable navigation system. Phys. Lett. A.

[2] Mougenot D., Thorburn N. (2004). MEMS-based 3D accelerometers for land seismic acquisition: Is it time?. Lead. Edge.

[3] Monajemi P., Ayazi F. (2006). Design optimization and implementation of a micro-gravity capacitive HARPSS accelerometer. IEEE Sens..

[4] Dong Y.G. (2007). Microsensor.

[5] King K., Yoon S.W., Perkins N.C., Najafi K. (2008). Wireless MEMS inertial sensor system for golf swing dynamic. Sens. Actuators A Phys..

[6] Nakamura S. (2005). MEMS inertial sensor toward higher accuracy & multi-axis sensing. Sensors.

[7] Sun C.M., Tsai M.H., Liu Y.C., Fang W. (2010). Implementation of a monolithic single proof-mass tri-axis accelerometer using CMOS–MEMS technique. IEEE Trans. Electron Devices.

[8] Ding H.G. (2006). Advances trends and recommendations in micro-nano technology. Nanotechnol. Precis. Eng..

[9] Tan X.Y., Liu X.W. (2004). Development of Small satellite and Micro-satellite Speed up By MEMS Technology. Chin. J. Sci. Instrum..

[10] Ruffin P.B., Burgetr S.J. (2001). Recent progress in MEMS technology development for military application. Proc. SPIE.

[11] Wu M.C., Solgaard O., Ford J.E. (2006). Optical MEMS for lightwave communication. J. Lightw. Technol..

[12] Xia S.H. (2001). Research and Development of Vacuum Microelectronic Sensors. J. Mech. Strength.

[13] Peng S.C., Wen Z.Y., Wen Z.Q., Pan Y.S., Li X. (2003). Finite Element Analysis on Vacuum Microelectronic Acceleration Sensor. Micronanoelectron. Technol..

[14] Paul J., Anthony J.K. (2009). A Planar CMOS Field-Emission Vacuum Magnetic Sensor. IEEE Trans. Electron Devices.

[15] Xu S.L. (2003). Study on Vacuum Microelectronic Pressure Sensor. Master’s Thesis.

[16] Wang B.P. (2002). Vacuum Microelectronics and Its Application.

[17] Neamen A.D. (2005). Semiconductor Physics and Devices.

[18] Liu H.T., Wen Z.Y., Shang Z.G., Chen L. (2014). A new method to analyze the stiffness of MEMS accelerometer. Key Eng. Mater..

